# Comparison between rasterstereographic scan and orthopedic examination for posture assessment: an observational study

**DOI:** 10.3389/fsurg.2024.1461569

**Published:** 2024-10-10

**Authors:** Samuel Weigel, Silvia Dullien, Joachim Grifka, Petra Jansen

**Affiliations:** ^1^ Department of Sports Sciences, University of Regensburg, Regensburg, Germany; ^2^ Regensburg University Medical Centre, Asklepios Klinikum, Bad Abbach, Germany

**Keywords:** postural abnormalities, orthopedic examination, rasterstereography, postural assessment, orthopedic assessment

## Abstract

**Background:**

Although the relationship between posture and back pain is still under debate, the potential role of body alignment highlights the importance of postural assessment in the health sector. Despite growing concern about musculoskeletal issues, there remains a lack of consensus on effective methods for detecting postural anomalies.

**Methods:**

This observational study compared postural assessments conducted by orthopedic specialists with those obtained through rasterstereographical spine scans using the DIERS formetric system. Fifty-four children from the third grade (mean age 9.4 years) underwent both assessments, allowing for a comprehensive examination of orthopedic abnormalities. Statistical analysis, including McNemar tests, was employed to compare the results of the assessments and evaluate potential discrepancies.

**Results:**

The comparison between the orthopedic examination and the DIERS scan revealed significant differences in assessing trunk imbalance (*p* < 0.001), thoracic kyphosis (*p* < 0.001), and lumbar lordosis (*p* < 0.001). Additionally, the study identified a high prevalence of orthopedic abnormalities, with 79.6% of the examined children exhibiting at least one issue in the orthopedic visual assessment.

**Conclusions:**

The study highlights the divergence between orthopedic evaluations and DIERS scans, emphasizing the challenges in achieving consistent postural assessments. The static analysis provided by the DIERS system, which quantifies posture in angles and distances, contrasts with the dynamic, functionality-focused approach of orthopedic examinations. The findings raise questions about the practicality and significance of integrating rasterstereography into routine pediatric orthopedic practice. The results underscore the complexity of postural evaluations and advocate for a comprehensive approach to address the multifaceted nature of back health in children.

## 1 Introduction

Upright posture is vital for physical health. Back problems, such as musculoskeletal issues, are widespread, leading to substantial healthcare costs (1). Among the younger population, this is already a significant concern. According to a survey by the German health insurance company DAK, 43% of students reported suffering from back pain, and 20% experienced it weekly (2). Additionally, the second wave of the German Health Interview and Examination Survey for Children and Adolescents (KiGGs), conducted from 2014 to 2017, showed a significant increase in the 3-month prevalence of back pain compared to the first wave of the survey from 2003 to 2006 (3). The prevalence of back pain increases as children grow older, reaching rates similar to those of adults (1, 4, 5). Postural abnormalities are also frequent during childhood (6, 7). While it is widely assumed that these abnormalities are connected to back pain, this has not been conclusively established. Studies have yielded mixed results regarding the relationship between body posture and back or neck pain, with some literature stating no such connection (8, 9). In children, poor posture was connected to headache and spinal pain in an observation in the Czech Republic (10). In females, pelvic position may contribute to the development of severe back pain (11). In addition, poor postural patterns may lead to reduced strength and endurance in the muscles that stabilize the spine (12), which could be a predictor for low back pain, as suggested by various studies (13, 14). Moreover, body posture has been found to predict hand, neck, and shoulder pain in a work setting (15, 16), while forward head posture also connects to neck pain in adults (16). In elderly populations, changed postural alignment has been associated with knee pain (17) and increased risk of falling in elderly females (18). Despite the unclear nature and mechanisms of the connection, postural analysis remains crucial in the health sector. Early identification of postural abnormalities is essential to prevent potential issues arising from these defects (19, 20).

Various evaluation methods, including x-rays, 3D MRI, spine scans, mobile applications, and orthopedic examinations, assess posture and identify orthopedic abnormalities (21–26). These technological assessment methods aim to reduce errors and optimize efficiency (27). By minimizing errors and ensuring high reproducibility, these assessment protocols should also be suitable for clinical and scientific applications (24).

The rasterstereography method is used to conduct spine scans. In this assessment, a light strip pattern is projected onto the back surface and captured by a camera. The software analyzes the line curvatures and employs photogrammetry to create a three-dimensional image of the back surface. Additionally, the 3D coordinates of the spine are calculated from specific landmarks using triangulation and are digitally displayed (28). Rasterstereography presents a valid and reliable analysis method for assessing the back surface and spinal parameters (29). This static analysis of various postural parameters quantifies posture regarding angles and distances (30). However, opinions are mixed regarding its potential to replace x-rays and reduce the need for repeated radiographs in high-precision posture analysis required to verify or monitor the progression of spinal deformities such as scoliosis (31–33). Some studies have shown variability between scans, possibly due to different positioning, breathing, or postural sway (29, 30, 34, 35). Additionally, Schroeder et al. (35) suggest that differences in soft tissue structure may influence the evaluation, particularly the positioning of landmarks used to assess pelvic position (34).

Traditional assessments performed by physicians are also widespread. In routine medical practice, initial assessments are conducted through visual evaluation, relying on the examiner's experience (36). Musculoskeletal evaluations by orthopedic specialists have shown high specificity for the early detection of abnormalities (37). These examinations assess many areas of the spine and lower extremities for their function, including spinal rotation, lateral inclination, and gait analysis. Such movements are integral to everyday life and allow the examiner to assess limitations in postural function. This is especially important as many back issues cannot be attributed to one specific anatomical abnormality in the spine (38). Some studies suggest a reduction in measurement reliability in lengthy assessments directly involving the subject, as in orthopedic examinations (39). This suggests that factors influencing orthopedic assessments may affect the subject and the examiner. While orthopedic knowledge is crucial for evaluating postural functionality, rater bias significantly influences posture assessments (40). Various studies have examined factors influencing the assessments of medical practitioners over time (41, 42). However, manual posture assessments by the same evaluator have proved reliable (43). Repeated exams by the same specialist allow the use of knowledge gained in previous exams to identify causes or propose treatments for detected issues (44). In children, this could include knowledge about the patient's growth, which can influence posture due to changes in body proportions (45).

Factors influencing the posture assessment conducted by orthopedic specialists may not necessarily affect more objective measurements, such as those obtained from the DIERS scan based on rasterstereography. Although the higher objectivity of rasterstereography supports its use for posture analysis, its application in everyday clinical practice is limited. According to Ludwig et al. (36), this method is rarely employed in many areas due to the high acquisition costs of the technological systems. Other research teams also scrutinize the benefit-cost relationship of rasterstereographical scans in everyday practice (46). Additionally, in clinical practice, objectively measured data still need to be interpreted by a specialist, thereby raising the dependence on the examiner's experience and the potential for rater bias.

There has been no direct comparison of postural assessment between visual orthopedic examinations and more technical diagnostic instruments, especially in young subjects. Therefore, this study aims to compare the postural assessment conducted by an orthopedic specialist with that provided by a rasterstereographical spine scan in elementary school children. Given the various factors that could influence the examiner's observations and the high objectivity of rasterstereographical scans, we anticipate differences in results, with the DIERS scan potentially identifying more orthopedic abnormalities.

## 2 Methods

### 2.1 Participants

The study was designed as an observational study. The study protocol was submitted in advance to the Ethics Committee of the University of Regensburg and received a favorable vote (No. 18-981-101). One hundred twenty children (the entire 3rd grade of the local primary school) were invited to participate in the study. The school was chosen due to its proximity to the examination site. Due to logistical and staffing constraints, the number of cases was limited to 60 children. Therefore, only the third grade was invited to participate to get a more homogenous sample (120 children total). The study management contacted parents who returned the invitation section with contact details, and appointments were scheduled at the Orthopedic Outpatient Clinic. There was no further selection from the potential participants after the invitation.

In the end, 54 children were examined, comprising 28 girls and 26 boys. Demographic characteristics of the patients are presented in Supplementary Table S1. Inclusion criteria were attendance in the third grade of the local Primary School, absence of acute illnesses or pain on the day of the examination, and a completed and signed consent form. None of the 54 willing participants had to be excluded.

### 2.2 Examination of the posture

The examination took place at the Orthopedic Outpatient Clinic. Upon arrival, parents were greeted with the information sheet and consent form. The medical examination and spinal measurements were conducted following the initial administrative steps.

#### 2.2.1 Orthopedic examination

The visual orthopedic examination, conducted by a specialist physician in orthopedics and traumatology and a senior physician in pediatric orthopedics, encompassed various assessments. From behind, the specialist inspected the back for abnormalities such as shoulder tilt, scapula winging, waist triangles, rib protrusion, possible lumbar bulge assessed through Adam's forward bend test, leg position including the measurement of the intercondylar and intermalleolar distances, foot position, and other irregularities. The sagittal view allowed documentation of the curvatures of the cervical, thoracic, and lumbar spine; frontal inspection involved assessing the shape of the thorax and gait. In the standing position, the physical examination covered single-leg stance, toe walk, heel walk, spinal tapping pain, kidney tapping pain, sacroiliac joint pressure pain, pelvic position, paravertebral muscle tone increase, muscle tone increases in the musculus trapezius, functionality of spinal rotation, lateral flexion of the spine and spinal extension, plumb line deviation, shoulder mobility, and finger-to-floor distance. The latter was measured as the distance between the floor, and fingertips were measured during maximum forward bending, with the knees kept straight. Therefore, no negative score was possible even with good flexibility, as the minimal score was zero. In the supine position, assessments included hip mobility, popliteal angle, which is a measure of the elasticity of the ischiocrural muscle (47), difference in leg length, muscle shortening of the musculus Quadrizeps femoris, its strength capabilities, and the Lasègue test (48).

After the examination, the doctor inquired about the presence of pain, its localization, if applicable, pain intensity (classified on a Visual Analogue Scale, VAS), and its triggers.

#### 2.2.2 Examination by video rasterstereography

The back scan was conducted using the DIERS formetric system (Schlangenbad, Germany), which employs video rasterstereography for postural analysis. This system has been scientifically validated and is often used for postural analysis (30, 34, 49, 50).

Five retroreflective adhesive markers (diameter 6 mm) were affixed to the children's unclothed backs to perform the back measurement, ensuring clear visibility of the course of the spinous processes. The posture was then measured in a relaxed, familiar position. The software utilizes various back and pelvis landmarks, along with these markers, to calculate parameters that quantify the posture and course of the spine. Parameters such as trunk inclination, trunk imbalance, pelvic tilt, pelvic torsion, kyphotic and lordotic angles, surface rotation, and lateral deviation are of particular interest. The postural parameters assessed with the DIERS formetric scan are described in detail in the Supplementary Table S2.

The postural measurements were then compared with reference values (51) to distinguish between normal and abnormal postural parameters. These values were derived from a study that conducted spinal scans with the formetric system in 497 fifth-grade school children in Germany. While alternative reference values for postural parameters exist (52, 53), these were assessed in adolescent and adult samples. Given that some posture parameters change with age (54), these references were not chosen for comparison in our young sample. Other existing reference values do not distinguish between normal and abnormal values; they provide only mean values for the measured postural parameters (55). Furthermore, the chosen values are cross-referenced within the software and manual of the DIERS system employed in this study, highlighting their suitability for comparison.

#### 2.2.3 Comparison between both examinations

**Figure 1:**
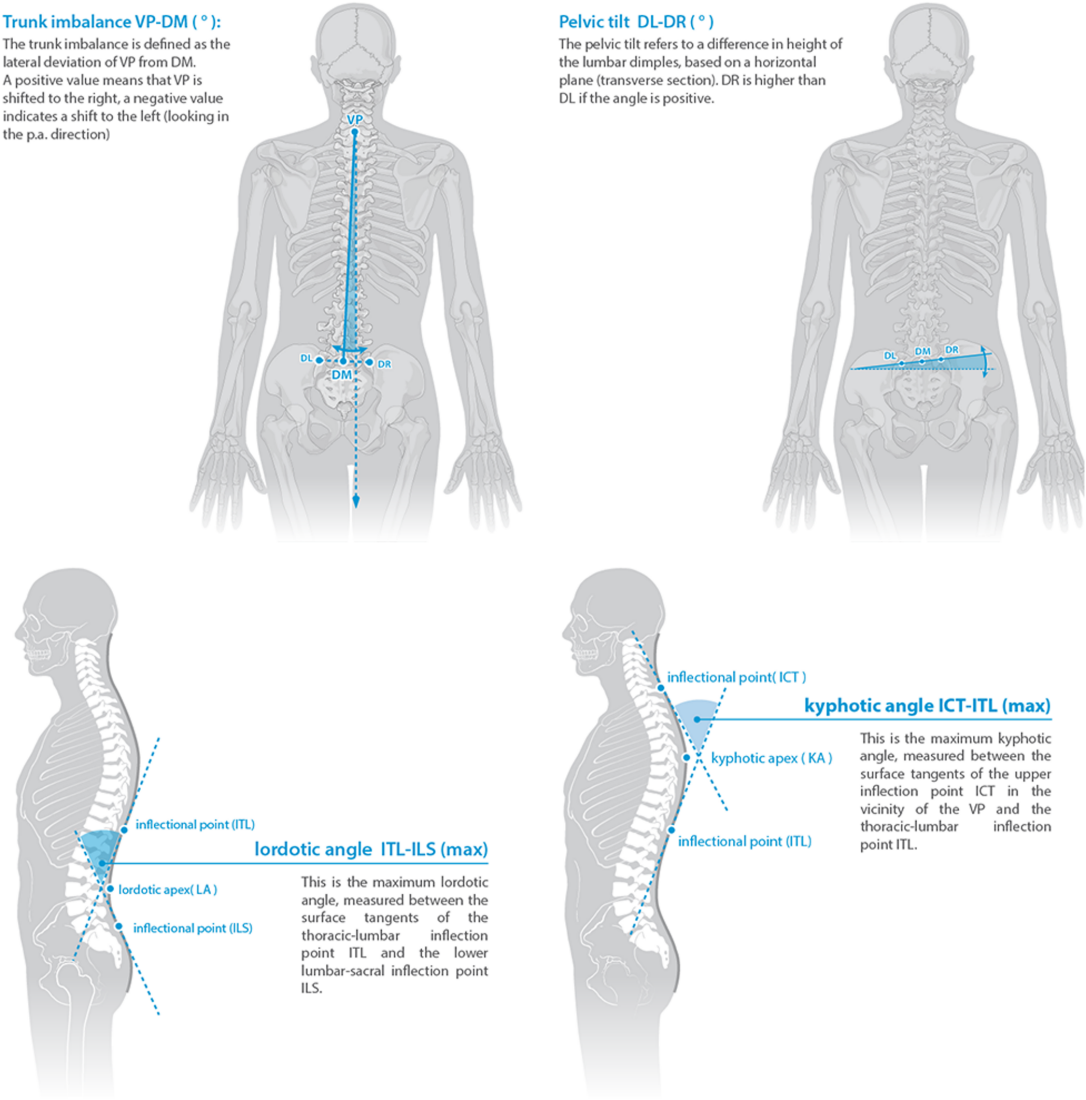
The four postural parameters compared between the two assessment methods. Reprinted from the DIERS formetric 4D website with permission (56).

Various posture parameters were examined in orthopedic evaluations and spine scan using the DIERS formetric system. The examination results for trunk imbalance and pelvic tilt can be directly compared in this process. Additionally, parameters providing information about the kyphosis and lordosis curvature of the spine are examined in both assessments. Figure 1 depicts which postural parameters were compared. The shown figures are taken from the DIERS formetric 4D website with permission (56). The orthopedic specialist also assessed the same postural parameters.

### 2.3 Statistical analysis

The statistical analysis was conducted using IBM SPSS Statistics 29 (IBM Inc., Chicago, IL, USA). Descriptive analyses were performed to determine incidences, mean values, standard deviations (SD), and range. The level of significance was set at *p* < 0.05. McNemar tests were conducted for each of the compared parameters to analyze the divergent evaluations made by the orthopedist and the DIERS-scan. This test is widely used to compare different diagnostic methods in paired samples with dichotomous observations, such as normal vs. abnormal, as assessed in the conducted posture evaluations and also if there is a relationship between the variable of interest (57). The examining orthopedic directly recorded the results of the assessments on an anonymized pen-and-paper examination form. These data were then compiled into an SPSS spreadsheet, along with the data exported from the formetric system.

## 3 Results

### 3.1 Abnormalities diagnosed by the orthopedic

The prevalence of different orthopedic abnormalities observed in the visual orthopedic examination is shown in Table 1.

**Table 1:** Prevalence of orthopedic abnormalities in the visual orthopedic assessment.

	Yes (*N* = ,% =)	No (*N* = ,% =)
Any orthopedic abnormality	43 (79.6%)	11 (20.4%)
Shoulder abnormalities	14 (25.9%)	40 (74.1%)
Upper body and spine abnormalities	20 (37.0%)	34 (63.0%)
Lower extremity abnormalities	27 (50.0%)	27 (50.0%)
Muscular abnormalities	15 (27.8%)	39 (72.2%)
Pain	5 (9.3%)	49 (90.7%)

Eleven (20.4%) of the 54 examined children showed no postural abnormalities in the visual orthopedic exam. Among the remaining 43 (79.6%) children, at least one of the examined characteristics was noticeable. The orthopedic abnormalities were retrospectively categorized into different groups, depending on the affected body region (see Supplementary Table S3). Abnormalities in the lower extremities were the most frequently examined; half (50.0%) of the sample displayed abnormalities in this area, which includes all issues related to the hips, legs, or feet. The assessment of pelvic tilt is included in this section, with one child (1.9%) having a noticeable pelvic position.

The second most prominent area of issues was the upper body, with 20 children (37.0%) displaying abnormalities. Most observed characteristics in this category were related to the shape and functionality of the spine, as it is a crucial part of the upper body concerning posture. Among other things, abnormalities in the compared parameters included trunk imbalance (0 children, 0%), thoracic kyphosis (2 children, 3.7%), and lumbar lordosis (1 child, 1.9%).

Fifteen children (27.8%) exhibited muscular abnormalities related to tension and shortening during the assessment. Only five children (9.3%) reported pain in response to various percussion tests or acute back pain.

In the measured clinical-orthopedic parameters, the mean popliteal angle was 11.8° on the right and 11.7° on the left. The mean finger-to-floor distance was 3.8 cm. Abnormalities in the leg axis were detected in only seven children (13%). The mean intercondylar and intermalleolar distances were 0.1 cm and 0.4 cm, respectively.

### 3.2 Abnormalities diagnosed by rasterstereography

The values assessed with the rasterstereography method are presented in Table 2.

**Table 2:** Measured parameters of the DIERS scan.

	Mean	SD	Range
Magnitude of trunk inclination [°]	2.4	1.9	0.2–8.8
Magnitude of trunk imbalance [mm]	7.5	8.1	0–23.4
Magnitude of pelvic tilt [mm]	3.5	3.0	0–15.0
Magnitude of pelvic torsion [°]	2.5	1.5	0–6.3
Kyphotic angle [°]	42.2	9.1	22.4–63.0
Lordotic Angle [°]	36.8	7.5	23.8–57.2
RMS Surface rotation [°]	4.3	3.0	1.0–15.6
Surface rotation amplitude [°]	9.0	3.8	2.9–22.8
RMS lateral deviation [mm]	4.0	2.1	1.2–11.8
Magnitude of maximum lateral deviation [mm]	7.2	3.1	2.5–18.1

RMS, root mean square.

As the direction of a possible deviation is interesting for this study, the magnitude of variances is reported for parameters with negative and positive values. A zero value indicates a balanced posture and is the goal. Some children showed perfect or near-perfect values in trunk inclination, trunk imbalance, pelvic tilt, and pelvic torsion. The lowest assessed magnitude for maximum lateral deviation was 2.5 mm. There were also children with high values for each parameter, resulting in a broad range.

For surface rotation, the mean amplitude was 9.0°, and the mean rms surface rotation was 4.3°. The mean values for the kyphotic and lordotic angles, describing the spinal curvature, were 42.2° and 36.8°, respectively. The mean rms lateral deviation was 4.0 mm.

Table 3 displays the count of identified abnormalities in the postural measurements, assessed by the reference values. One (1.9%) child showed no postural abnormality in the rasterstereographical scan.

**Table 3:** Posture rating according to reference values (51).

	Reference value	Abnormality assessed
Trunk inclination [°]	0–3	9 (16.7%)
Trunk imbalance [mm]	−10–10	17 (31.5%)
Pelvic tilt [mm]	−10–10	1 (1.9%)
Pelvic torsion [°]	−3–3	18 (33.3%)
Kyphotic angle [°]	45–55	43 (79.6%)
Lordotic angle [°]	Boys: 32–37 Girls: 40–45	42 (77.8%)
RMS surface rotation [°]	0–5	15 (27.8%)
Surface rotation amplitude [°]	−8–8	30 (55.6%)
RMS lateral deviation [mm]	0–5	4 (7.4%)
Maximum lateral deviation [mm]	−10–10	6 (11.1%)

### 3.3 Comparison between both measurements

Table 4 depicts the different ratings of the compared postural parameters. The percentage refers to the share of the whole sample in which the ratings differed or only one assessment method found an abnormality. The statistical power and the effect size are also presented. The effect size *w* was calculated according to Steyn (58).

**Table 4:** Difference in postural assessments between orthopedic assessment and rasterstereographical scan.

Postural parameter	Diverging ratings (*N* = ,% =)	Abnormality found only in orthopedic assessment (*N* = ,% =)	Abnormality found only in rasterstereographical scan *N* = ,% =)	Significance level of difference	Effect size (*w* =)	Statistical power
Trunk imbalance	17 (31.5%)	0 (0%)	17 (31.5%)	*p* < 0.001	0.94^a^	0.79
Pelvic tilt	2 (3.7%)	1 (1.9%)	1 (1.9%)	*p* > 0.05	0	0
Thoracic kyphosis	41 (75.9%)	2 (3.7%)	39 (72.2%)	*p* < 0.001	0.88^a^	>0.99
Lumbar lordosis	38 (70.4%)	1 (1.9%)	37 (68.5%)	*p* < 0.001	0.94^a^	0.99

^a^ This indicates a large effect according to (58).

## 4 Discussion

### 4.1 Differences in postural assessment

Assessing the mentioned postural abnormalities with different methods resulted in divergent results. We can only speculate about the reasons. One reason might be the influence of posture during the test. The orthopedic examination was done by two senior orthopedic practitioners, whose experience reduces a possible variability in the examination. However, the raster stereography was interpreted by a sports scientist with less experience than the orthopedic examiners. In the study with adults, the reliability of the DIERS System is good (22).

These diverse assessment criteria restrict the direct comparison to only a few parameters. The assessment of spinal curvature revealed the highest difference in ratings, with over 60% differences in the evaluation of kyphosis and lordosis. This dissimilarity can be attributed to the distinct nature of each assessment method. Studies indicate that lumbar angles vary in different positions (59). This phenomenon was also noted in various postural parameters when transitioning from a habitual to an active posture in a sample of male adolescents (60). In both assessments in our study, participants were instructed to assume a relaxed, normal standing position. However, slight variations in posture could lead to different measurements and ratings, potentially contributing to the high rate of disagreement in these assessments.

Furthermore, the importance of precise placement and correct positioning in examinations using the DIERS formetric system is emphasized (30) to obtain accurately and correctly measured postural parameters. Slightly differing placements could also account for some differing measurements. For this, it is important to implement quality control. The DIERS system's scientists must establish a learning curve while performing the diagnostic, which has shown to reduce variances between repeated raterstereographic scans (30). This learning curve must be monitored. For this, a longer practice with rasterstereography is necessary, especially in pediatric orthopedics, where the children might show more variability. Only a few studies support the validity of rasterstereography in detecting spinal disorders. A small number of participants limits most and they focus only on adolescent idiopathic scoliosis, neglecting other abnormalities (33). Some research teams believe that, when executed with precision, this method facilitates rapid and non-invasive data collection, making it appropriate for the initial assessment of postural abnormalities (27, 30). A meta-analysis demonstrated high validity and reliability for the postural parameters of lumbar lordosis and thoracic kyphosis—both assessed and compared in our study—when comparing a DIERS scan to the gold x-ray standard (61). The rasterstereographic scan using the Formetric system demonstrated high sensitivity and good specificity in assessing postural abnormalities when applying the reference values described in the study conducted by Harzmann (51), which emphasized the importance of having an experienced diagnostician conduct the scan. However, since no comparison was made with the gold standard of x-rays, it was impossible to calculate sensitivity and specificity in our study. In contrast, other authors have described these reference intervals as too narrow, suggesting a broader spectrum of values should be considered normal, particularly regarding kyphotic and lordotic angles (62, 63). This strict classification could potentially result in healthy children being diagnosed with postural abnormalities, leading to false positives in the rasterstereographic scan.

Given that most of the abnormalities identified by the DIERS scan were close to the reference ranges, this could explain the high number of abnormalities detected by the Formetric system. Expanding the reference frame by 50% of the original width in both directions reveals many elevated values within these extended boundary areas. For trunk imbalance, 14 of the 17 assessed abnormalities are within 10 mm of the reference borders. Similar trends are observed for the lordotic and kyphotic angles, where 14 of 42 and 16 of 43 findings are relatively close outside the used reference values, respectively. When applying the widened reference interval for spinal angles, 13 children (24.1%) showed normal values, compared to only 2 children (3.7%) when classified using the narrower reference range. Many of these cases, with values near the border of the normal range, were simultaneously assessed as medically unremarkable by the orthopedic examiner. For trunk imbalance, this applies to all 14 children. For the lordotic angle in these border areas, 12 cases were not seen as striking in the visual observation, and for the kyphotic angle, 14 cases were not considered abnormal.

In contrast to the spine scan, orthopedic practitioners can integrate their knowledge about abnormal postural patterns during the assessment (64). This integration becomes evident in the form of rating abnormalities that might not be detected during the brief assessment in the scanner. Simulated postures can greatly impact the short observation duration in the rasterstereographical scan, even if they occur subconsciously. However, the child's genuine standing posture during the orthopedic visual assessment may be incorporated into the specialist's rating.

The question arises whether the application of rasterstereography in daily pediatric orthopedic practice is both practical and meaningful, or if the conventional approach of functionality-focused postural assessments conducted by orthopedic specialists or pediatricians suffices. Another method to analyze the posture more indirectly by load distribution on the feet and gait dynamics is the use of baropodometry. For example, in a pilot study with children suffering from scoliosis, it has been shown that they have different loading patterns than children from a healthy control group (65).

### 4.2 Found orthopedic abnormalities

The elevated prevalence of orthopedic abnormalities identified through both assessment methods corresponds with findings from other studies, highlighting many postural issues in children (6, 10, 66). The mean popliteal angle was lower in the observed sample compared to the results of other studies, which report mean values around 25° in children this age group. The observed children, therefore, showed a higher hamstring flexibility than the reference groups (67, 68). The measured intercondylar and intermalleolar distances are low, as the literature suggests a classification as abnormal when distances are more than 5 cm and 7 cm, respectively (69).

However, challenges arise when interpreting identified abnormalities as reliable indicators of future issues. Various studies on changes in posture throughout childhood and adolescence present divergent results, indicating either preconditions for future postural problems or normal side effects of different growth phases (6, 55, 70, 71).

### 4.3 Limitations

One limitation of this study is the small sample size of 54 participants, due to logistical and personnel constraints. The non-mobile examination technique necessitated that the selected school be close to the examination site. Additionally, the limited number of postural parameters available for comparison is another constraint, arising from the differing focus points of the two evaluation methods. Specifically, the standard postural examination conducted by the orthopedic examiner at the clinic influenced the parameters available for comparison.

### 4.4 Conclusion

Orthopedic, postural assessments and rasterstereographical spine scans presented divergent results, with spine scans identifying more postural abnormalities in the compared parameters. This raises questions about the choice of general practice and the cost-benefit ratio of different methods in pediatric postural assessment. Influencing factors can occur in both methods during observations or the interpretation of the results. Visual orthopedic assessments offer a quick and cost-efficient observation of posture. The reliance on the examiner can be positive, as experience-based evaluation can enhance the assessment. However, this also results in more subjective interpretations and potential rater bias. Conversely, even the objective data from spine scans are interpreted by human practitioners, introducing possible subjective influencing factors.

Regardless of the parameters compared, the prevalence of orthopedic abnormalities in children was high in both methods, consistent with existing literature. The interpretation of these findings remains disputed, whether as normal byproducts of growth phases or as indicators of future postural problems.

## Data Availability

The original contributions presented in the study are included in the article/Supplementary Material, further inquiries can be directed to the corresponding author.
