# Supplementary material for: Comparison between rasterstereographic scan and orthopedic examination for posture assessment: an observational study
Source: Front Surg. 2024 Oct 10;11:1461569. doi: 10.3389/fsurg.2024.1461569 (PMC11499226; doi:10.3389/fsurg.2024.1461569)
Supplement: Supplementary file 1 [file Table1.docx]

# Demographic characteristics of the patients

|  | **N** | **Mean** | **SD** | **Range** |
| --- | --- | --- | --- | --- |
| Male | 26 |  |  |  |
| Female | 28 |  |  |  |
| Age [years] | 54 | 9.4 | 0.4 | 8.7 – 10.4 |
| Heigth [m] | 54 | 1.38 | 0.05 | 1.28 – 1.49 |
| Bodyweight [kg] | 54 | 32.3 | 5.8 | 21.8 – 54.5 |
| BMI [kg/m^2^] | 54 | 16.8 | 2.3 | 12.2 – 24.7 |
