# Supplementary material for: Comparison between rasterstereographic scan and orthopedic examination for posture assessment: an observational study
Source: Front Surg. 2024 Oct 10;11:1461569. doi: 10.3389/fsurg.2024.1461569 (PMC11499226; doi:10.3389/fsurg.2024.1461569)
Supplement: Supplementary file 2 [file Table2.docx]

## Description of the parameters assessed in the DIERS-scan

| Trunk Inclination (VP-DM) | Angle in degrees between the plumb line of the vertebra prominens (VP) and the line connecting the VP to the center of the lumbar dimples (DM). Negative values indicate trunk backward inclination, positive values indicate trunk forward inclination. |
| --- | --- |
| Trunk imbalance (VP-DM) | Lateral distance of the plumb line (positioned at VP) to the center of the dimples, positive value means VP is shifted to the right. |
| Kyphotic Angle (ICT-ITL) | Angle between the surface tangent at the cervico-thoracic inflection point (ICT) and the surface tangent at the thoracic-lumbar inflection point (ITL) in degrees. |
| Lordotic Angle (ITL-ILS) | Angle between the surface tangent at the thoracic-lumbar inflection point (ITL) and the surface tangent at the lumbar-sacral inflection point (ILS) in degrees. |
| Pelvic Tilt (DL-DR) | Height difference of the lumbar dimples (DL & DR), positive values mean the right side is higher. |
| Pelvic Torsion (DL-DR) | Reciprocal torsion of the surface normals on the two lumbar dimples (DL & DR), positive values mean right side is twisted forward. |
| Surface Rotation (rms) | The root mean square of the horizontal component of surface normals on the symmetry line. Notation: A positive value of surface rotation describes rotation to the right (spinous process points to the right). |
| Surface Rotation (Amplitude) | This value describes the maximum twist of the spine and is calculated as the sum of the magnitudes from the rotation to the left and right. |
| Lateral Deviation (max) (VP-DM) | The maximum deviation of the midline of the spine from the direct connection VP-DM in the frontal plane. |
| Lateral Deviation (rms) (VP-DM) | The root mean square of the deviation of the midline of the spine from the direct connection VP-DM in the frontal plane. |
