# Supplementary material for: Comparison between rasterstereographic scan and orthopedic examination for posture assessment: an observational study
Source: Front Surg. 2024 Oct 10;11:1461569. doi: 10.3389/fsurg.2024.1461569 (PMC11499226; doi:10.3389/fsurg.2024.1461569)
Supplement: Supplementary file 3 [file Table3.docx]

## Classification of the observed abnormalities in the orthopedic examination

| **Summarized concept** | **Included abnormalities** |
| --- | --- |
| Shoulder abnormalities | - Shoulder tilt - Scapula alata - Shoulder mobility |
| Upper body and spine abnormalities | - Waist triangle - Rib hump - Lumbar roll - Cervical lordosis - Thoracic kyphosis - Lumbar lordosis - Thoracic shape - Spinal rotation - Spinal lateral tilt - Spinal reclination - Plumb line deviation |
| Lower extremity abnormalities | - Leg axis - Foot position - Knee axis - Gait pattern - Single-leg stance - Toe walking - Heel walking - Pelvic tilt - Hip mobility - Leg length discrepancy |
| Muscular abnormalities | - Increased paravertebral muscle tone - Trapezius muscle tone increase - Quadriceps muscle shortening - Hamstring muscle shortening - Strength assessment |
| Pain | - Spinal percussion tenderness - Kidney percussion tenderness - Sacroiliac joint tenderness - Acute back pain - Localization of back pain - Triggers of back pain - Positive Lasègue test |
